# The Proliferating Cell Nuclear Antigen (PCNA) Transcript Variants as Potential Relapse Markers in B-Cell Acute Lymphoblastic Leukemia

**DOI:** 10.3390/cells11203205

**Published:** 2022-10-12

**Authors:** Vanessa Villegas-Ruíz, Antonio Romo-Mancillas, Isabel Medina-Vera, Kattia Alejandra Castro-López, Josselene Carina Ramirez-Chiquito, Marco Antonio Fonseca-Montaño, Mercedes Edna García-Cruz, Roberto Rivera-Luna, Julieta Griselda Mendoza-Torreblanca, Sergio Juárez-Méndez

**Affiliations:** 1 Experimental Oncology Laboratory, National Institute of Pediatrics, Mexico 04530, Mexico; 2 Computer Aided Drug Design and Synthesis Laboratory, School of Chemistry, Autonomous University of Querétaro, Queretaro 76010, Mexico; 3 Research Methodology Department, National Institute of Pediatrics, Mexico 04530, Mexico; 4 Neuroscience Laboratory, National Institute of Pediatrics, Mexico 04530, Mexico; 5 Department of Pediatric Oncology, National Institute of Pediatrics, Mexico 04530, Mexico; 6 Molecular Pathology Laboratory, Department of Pathology, National Institute of Pediatrics, Mexico 04530, Mexico

**Keywords:** B-ALL, leukemia, PCNA, alternative splicing

## Abstract

Leukemia is the most common childhood malignancy in Mexico, representing more than 50% of all childhood cancers. Although treatment leads to a survival of up to 90% in developing countries, in our country, it is less than 65%. Additionally, ~30% of patients relapse with poor prognosis. Alternative splicing plays an important role in transcriptome diversity and cellular biology. This mechanism promotes an increase in the assortment of proteins with potentially distinct functions from a single gene. The proliferating cell nuclear antigen (PCNA) gene encodes two transcripts for the same protein of 261 amino acids, which is associated with several important cellular processes and with several types of cancer. However, the diversity of the transcript variants expressed in this condition is not clear. Then, we used microarray gene expression to identify changes in the exon expression level of PCNA. The data were validated using RT-PCR and Sanger sequencing, and three additional transcripts (PCNA_V3, PCNA_V4, and PCNA_V5) were identified. Computational analyses were used to determine the potential proteins resulting, their structure, and interactions with PCNA native protein and themselves. Additionally, the PCNA transcript variants were inhibited using specific siRNA, determining that their inhibition contributes to the malignant characteristics in vitro. Finally, we quantified the PCNA transcript variants in acute lymphoblastic leukemia samples and identified their expression in this disease. Based on the clinical characteristics, we determined that PCNA_V2 and PCNA_V4 are expressed at significantly low levels in relapsed B-ALL patients. We conclude that the low expression of PCNA_V2 and PCNA_V4 could be a potential molecular marker of relapse in acute lymphoblastic leukemia patients.

## 1. Introduction

Leukemia is the most common childhood malignancy worldwide; in Mexico, leukemia represents more than 50% of childhood cancers. Although treatment leads to a survival up to 90%, in developing countries, in our country, it is less than 65% [1]. Additionally, leukemia showed several alterations, including chromosomal and genetic aberrations and deregulation of protein and gene expression. Although the treatment of leukemia patients in Mexico is systematic, based on several clinical characteristics and classification of risk groups, ~30% of the patients present relapse [2]. The molecular and cellular characteristics that promote relapse are not completely clear. However, several genetic abnormalities are very important for prognosis, such as ETV6-RUNX1 [3] and BCR-ABL [4], among others, but these abnormalities are present in a low number of patients.

Alternative mRNA splicing plays a central role in transcriptome diversity and, consequently, in the generation of multiple proteins with potentially distinct functions from a single gene [5]. There is not a complete sequence of the human transcriptome. However, it has been suggested that the relationship between genes and transcripts is ~1:10. Multiple combinations of alternative splicing (AS) result in very difficult identification of potential alternative mRNA transcripts by bioinformatic analysis. Therefore, high-throughput technologies such as microarrays and next-generation sequencing have provided an accelerated understanding of transcriptome diversity.

Aberrant AS is very common in cancer, and this condition could be used to find specific biomarkers for this disease. However, the great challenge is the identification of the specific molecule that has an important impact on diagnosis, prognosis and treatment in several human malignances. Currently, diverse protein products of AS have been associated with several types of cancer, such as BCL-XL (anti-apoptotic protein) [6], RON [7], VEGF-A [8], and TP53 [9].

The proliferating cell nuclear antigen (PCNA) gene is located in the negative chain on chromosome 20p12.2. The protein encoded by this gene is found in the nucleus, and its function is mediated by DNA and protein interactions. The encoded protein acts as a homotrimer and has been associated with several cellular processes, including DNA replication [10,11], DNA repair [12] and chromatin remodeling [13]. PCNA has two alternative transcripts reported in the NCBI and Ensembl databases. The large transcript contains seven exons (ENST00000379160.3, NM_002592.2), and the short transcript contains six exons (ENST00000379143.10, NM_182649.2). Although there are differences in the 5′ end of both transcripts, the resulting protein is the same, with 261 amino acids. Moreover, PCNA is overexpressed in several types of cancer. However, the diversity of the transcript variants expressed in this condition is not clear. Then, the aim of this work was to characterize the structure and function of novel PCNA transcript variants to find a potential molecular maker of relapse in acute lymphoblastic leukemia.

## 2. Materials and Methods

### 2.1. Microarray Data Mining

The microarray data were obtained from ArrayExpress and Gene Expression Omnibus (GEO). We selected data from B-cell acute lymphoblastic leukemia (B-ALL), healthy control B cells (B Cell) and B-ALL cell lines (B-CL) (Table 1). The microarray data were selected for quality control, and the microarray analysis was performed using Partek Genomics Suite version 6.6 according to previous studies [14,15,16,17,18,19]. The significant differences between B-ALL, B-CL, and B-Cell were obtained using ASANOVA with an FDR *p* value < 0.005.

### 2.2. Sample Acquisition

In this work, we included 70 patients diagnosed with B-ALL and 20 healthy peripheral blood controls. We obtained bone marrow at diagnosis, with previously signed informed consent from the patients, and peripheral blood of the healthy controls. The protocol was approved by the Institutional Ethics Committee (INP protocol 060/2016) CONBIOÉTICA-09-CEI-025-20161215, in accordance with the Declaration of Helsinki. The bone marrow and peripheral bone were treated to obtain the cell pellets as well as RNA according to previous studies [15,20].

### 2.3. RNA Purification

Total RNA was purified using an RNAeasy Mini Kit (Qiagen, Valencia, CA, USA) according to the manufacturer’s instructions and previous reports [15,19,20]. The total RNA concentration was quantified using a NanoDrop One UV-Vis Spectrophotometer (Thermo Fisher Scientific, Waltham, MA, USA). Total RNA was stored at −70 °C until use.

### 2.4. Culture Cell Lines

The RS4 and HTR-8/SVneo cell lines were cultured in Roswell Park Memorial Institute (RPMI) 1640 Medium (Gibco, Waltham, MA, USA) at 37 °C and 5% CO_2_ supplemented with 10% fetal bovine serum and 1% penicillin-streptomycin (Gibco, Waltham, MA, USA).

### 2.5. RT-PCR and Sequencing

One microgram of total RNA was digested with DNase after cDNA synthesis according to previous studies [15,20]. PCR amplification was performed using 25 ng of synthesized cDNA and Fast HotStart DNA Polymerase KAPA2G, (Kapa Biosystems, Wilmington, MA, USA). We used the RPL4 housekeeping gene according to a previous report [20] and several pairs of primers to amplify PCNA transcript variants, as shown in Table S1.

### 2.6. Rapid Amplification of cDNA Ends 3′ RACE

The 3′ RACE procedure was performed using a 3′ RACE System for Rapid Amplification of cDNA Ends Kit (Thermo Fisher Scientific, Waltham, MA, USA) according to a previous report [20]. We performed nested PCR using the primer pairs shown in Table S1. The PCR products were separated by means of 2% agarose gel electrophoresis and purified using a Zymoclean Gel DNA Recovery Kit (Zymo Research, Irvine, CA, USA) according to the manufacturer’s protocol. After that, the PCR products were sequenced as described.

### 2.7. Homology Modeling

The homology models of the sequences of the PCNA variants (variants 3 and 4) were built using the Robetta web server [21], which performed a search for homologous sequences to, later, build the model. The structural information of the native structure of PCNA V1/2 was obtained from the UniProt database (PDB: 1U7B) [22]. Once the three-dimensional structures of the variants and the crystallography were obtained, they were visually reviewed in Schrödinger-Maestro 2017-4 and prepared for further studies using Protein Preparation Wizard [23] available in the Maestro suite.

### 2.8. Molecular Dynamics

To evaluate the stability of the models and their behavior compared to the native structure, molecular dynamics simulations were performed under different conditions. Data analyses were performed in R and PyMOL 2.0 for image generation.

### 2.9. Atomistic Simulations (All-Atom)

To assess the stability of the variant models, they were subjected to 50 ns of molecular dynamics simulations using gromacs 2018.7 [24,25,26,27,28]. Briefly, the structures were properly prepared by adding model water TIP4PEW and ions at a total concentration of 0.1 M, then they were minimized and subjected to several equilibrium processes with restriction of positions of the peptide bonds of the proteins in different assemblies (500 ps NVT, 4 × 1 ns NPT). Finally, the system was subjected to a production step of 50 ns at 310 K and 1 bar in an NPT assembly using temperature coupling and velocity rescaling with stochastic term [29] as a thermostat and the Parrinello–Rahman barostat [30].

### 2.10. Simplified Simulations (Coarse-Grained)

To evaluate the behavior of the models with their interaction with DNA, a 50 bp sequence was constructed in the Web3DNA2.0 web server [24], whose sequence is a repetition of the one reported in a recent PCNA crystallography (PDB:6GIS) [25]. This optimized DNA fragment, using reference crystallography, was inserted into the different PCNA templates obtained by homology modeling. These systems were converted into a coarse-grained model using the Martini force field [26,27] for DNA [28], the ElNeDyn algorithm [29] to consider protein flexibility, and a polarizable water model [30]. The prepared systems were subjected to dynamics simulations of 1 μs using gromacs 2018.7 using the same assembly conditions, pressure and temperature used in the atomistic simulations. The resulting structures were converted to their atomistic structure using Backward [31] to perform the relevant analyses.

### 2.11. Quantitative RT-PCR

Quantitative RT-PCR was performed using 10 ng of cDNA, and we performed a mix that contained KAPA SYBR FAST qPCR Master Mix Universal 1X (Kapa Biosystems, Wilmington, MA, USA), forward and reverse primers 0.2 μM, and ROX High Reference Dye 1X (Kapa Biosystems, Wilmington, MA, USA). Quantitative RT-PCR was performed on a Step One Real-Time PCR System (Applied Biosystems Inc., Foster City, CA, USA). The primer pairs are shown in Table S1. The reaction mixture was incubated for 10 min at 95 °C and then 30 cycles for amplification for 15 s at 95 °C and 30 s at 60 °C. Finally, the melting curve was obtained at 50 °C for 1 min with quantification every +0.3 °C for 15 s up to 95 °C.

### 2.12. Droplet Digital PCR

Droplet digital PCR was performed using QX200 ddPCR Eva Green Super Mix (Bio-Rad, Hercules, CA, USA) in a QX200 Droplet Digital PCR system (Bio-Rad). The protocol was performed according to a previous report [15]. ddPCR was performed using the same primers used for quantitative RT-PCR. All experiments were normalized to 100 copies of the RPS18 housekeeping gene.

### 2.13. RNA Interference

We employed an on-target siRNA, scrambled (cat. D-001810-01-20), On-TARGETplus PCNA_V3 5′ P.A.C.U.C.C.U.G.U.U.C.C.U.G.U.G.A.G.G.U. U 3′, On-On-TARGETplus PCNA_V4 5′ P.U.U.U.G.G.A.C.A.U.A.C.U.U.C.U.U.G.A.G.G. A 3′ (GE Healthcare Dharmacon, Inc. Lafayette, CO, USA), and the positive control was a Mimic Housekeeping Positive control (GAPDH, cat. CP-001000-02). The transfection was performed in six-well plates, and we used 3.5 × 10^5^ cells and carried out the experiments in triplicate. After 24 h, the culture medium was replaced with a mixture containing 7.5 µL of Lipofectamine 3000 (Thermo Fisher Scientific, Waltham, MA, USA) and 50 nM of siRNA up to 2 mL of the final volume with Opti-MEM (Thermo Fisher Scientific, Waltham, MA, USA). Twenty-four hours after transfection, we reestablished the RPMI medium. Finally, the total RNA was purified using RNeasy kit (Qiagen, Hilden, Germany). The total RNA was stored at −70 °C until use, and cDNA synthesis was performed according to a previous method.

### 2.14. Cell Viability

Cell viability was measured using MTT (3-(4,5-dimethylthiazol-2-yl)-2,5-diphenyltetrazolium bromide) (Thermo Fisher Scientific, Waltham, MA, USA). The assay was performed in 96-well plates, and 4000 cells were collected per well. After 24 h of cell growth, the cells were washed with 1× PBS and treated with 50 µg/µL MTT in each well. The plate was incubated for four hours at 37 °C protected from light. Then, we solubilized the formazan using 100 μL of DMSO by pipetting. Then, the plate was incubated for 10 min at 37 °C. Finally, the plate was homogenized for fifteen minutes in a level four Titer plate shaker (Thermo Fisher Scientific, Waltham, MA, USA) and then read in a SPECTRA max Plus to 570 nm (Molecular Devices, San Jose, CA, USA).

### 2.15. Transwell Migration Assay

The transfected cells were transferred to a transwell chamber at 24 h posttransfection, and cells in the logarithmic phase were digested and incubated in RPMI-1640 medium free of FBS. The micropore membrane of the Transwell chamber was 8 µm (Corning Inn., New York, NY, USA). The lower chamber was filled with RPMI-1640 supplemented with 10% FBS, whereas the upper transwell chamber was filled with 200 μL of the 10,000 suspension cells according to the technical specifications of Corning. After 24 h, the membrane was fixed using 4% paraformaldehyde and stained with 0.1% crystal violet. The cells on the surface of the upper chamber were removed using cotton swabs. The experiments of each group were repeated three times. The cell number was counted as follows: The fractionator method was employed to estimate the total cell number at the bottom of the net without bias. Through a counting frame and systematic random sampling, the number of cells was extrapolated by using the following formula:

$$\sum Q-.\;\frac{1}{asf}.\;\frac{1}{ssf}$$

where Ʃ*Q*− = Cells counted; *asf* = Area sampling fraction; *ssf* = Section sampling fraction. Area sampling fraction = Grid size/Counting frame area; Section sampling fraction = 1 (because the counting was in a flat region). The grid size was 850 × 650 µm, and the counting frame size was 330 × 200 µm. The estimated total number of cells was calculated using the Stereo Investigator 9 program in a semiautomatic stereological system (MicroBrightField Inc., Williston, VT, USA).

### 2.16. Statistical Analysis

One-way ANOVA with Dunnett’s post-hoc analysis was used to assess the mRNA inhibition of PCNA transcript variants and cell migration. The Mann-Whitney U test was used to compare the gene expression (PCNA_V1, PCNA_V2, PCNA_V3, and PCNA_V4) between the control group and the B-ALL group and for the association between gene expression (PCNA_V1, PCNA_V2, PCNA_V3, and PCNA_V4). Pearson coefficients were calculated and displayed in a heatmap. Differences in the expression of the PCNA_V2/RPS18 and PCNA_V4/RPS18 transcripts between patients with relapse and without relapse were evaluated using the Mann-Whitney U test. Receiver operating characteristic (ROC) curves were generated, as were the area under the curve (AUC) and 95% confidence interval (95% CI). The cutoff point for PCNA_V2 was 266.8 RNA copies while for PCNA_V4, it was 87.74 RNA copies, were determined using the Youden index (Y = sensitivity + specificity − 1), taking the value closest to 1. Finally, the Kaplan-Meier method was used to estimate relapse-free survival (RFS) with analysis of differences using the log-rank test, and hazard ratios (HRs) were calculated using Cox proportional hazards. Finally, one-way ANOVA with Dunnett post hoc analysis was used to assess PCNA_V3 and PCNA_V4 expression in 25 cancer cell lines by quantitative RT-PCR. A value of *p* < 0.05 was considered statistically significant. Data were analyzed using the Statistical Package for the Social Sciences version 25.0 (SPSS Inc., Chicago, IL, USA).

## 3. Results

### 3.1. Proliferating Cell Nuclear Antigen Expressed Novel Transcripts Variants in B-Cell Acute Lymphoblastic Leukemia

**Figure 1:**
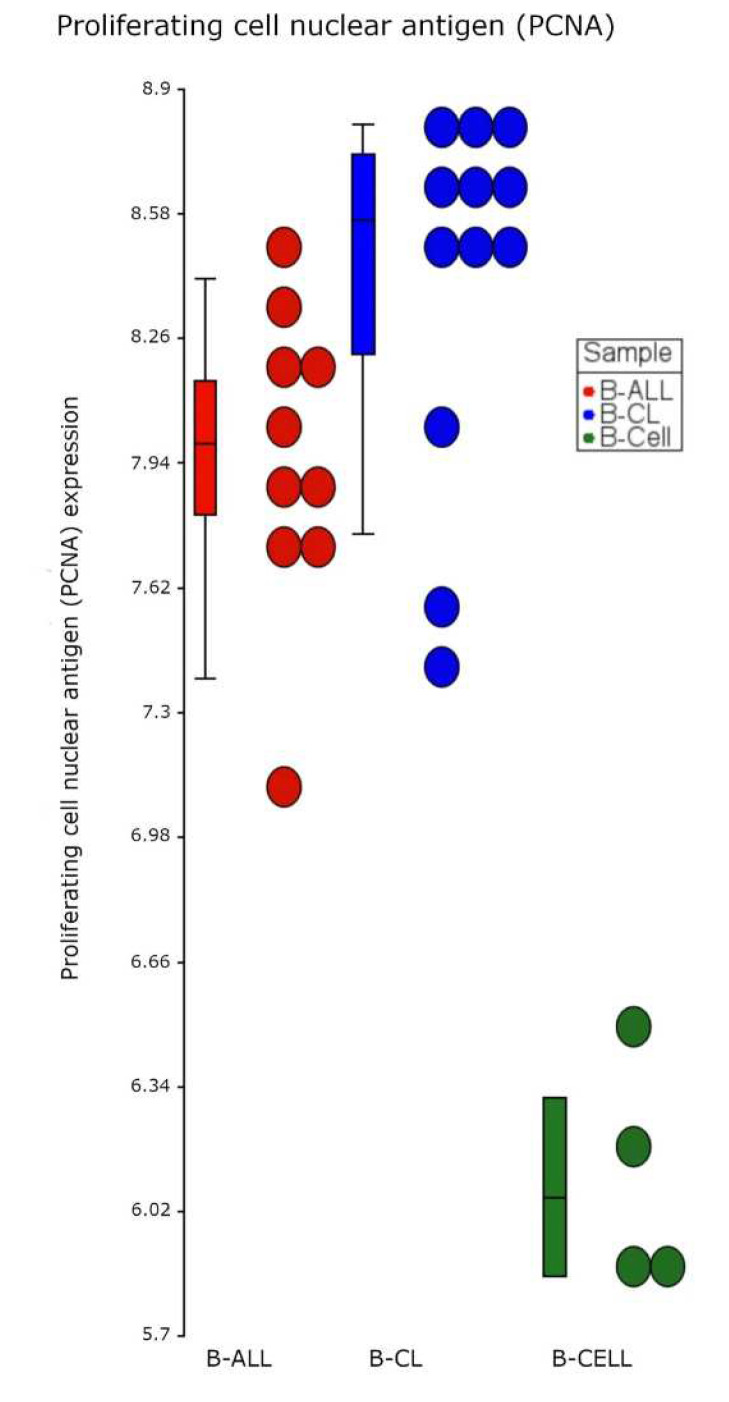
Dot plot expression of proliferating cell nuclear antigen (PCNA) in B-cell acute lymphoblastic leukemia and cell lines. The plot shows the level of PCNA gene expression in B-ALL cells from patients (red circles), B-ALL cell lines (B-CL, blue circles), and healthy B cells (B cells, green circles). We observed significant overexpression of PCNA in B-ALL and B-CL cells (FDR value = 4.23–54 and fold change 3.64) using ASANOVA.

**Figure 2:**
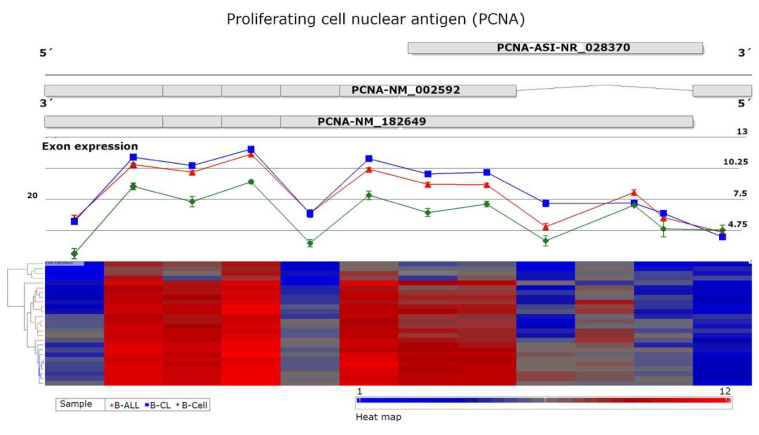
Exon expression profile of the proliferating cell nuclear antigen (PCNA) gene. The top of the figure shows possible splice variants of PCNA (PCNA-NM_002592; PCNA-NM_182649) in the antisense DNA chain and the sense noncoding DNA chain (PCNA-ASI-NR_028370). The middle of the figure depicts the mean of individual exon expression in red (B-cell acute lymphoblastic leukemia), blue (B-cell acute lymphoblastic leukemia cell lines), and green (healthy B cells). At the bottom is a heatmap of each exon level expression; the red color indicates high expression, and blue color indicates low expression. Significant differences in exon level expression were observed using ASANOVA with FRD value = 6.49^−18^.

The microarray gene expression profile of B-ALL-associated cells showed that PCNA (RefSeq, NM_002592) was significantly overexpressed in B-ALL patients and B-ALL cell lines, FDR *p* value = 4.23^−54^ and fold change 3.64, as shown in Figure 1. Additionally, we analyzed the exon expression profile and identified significant differences (FDR *p* value = 6.49^−18^). Our results indicate that PCNA is upregulated and expresses other transcript variants that have not been identified (Figure 2).

**Figure 3:**
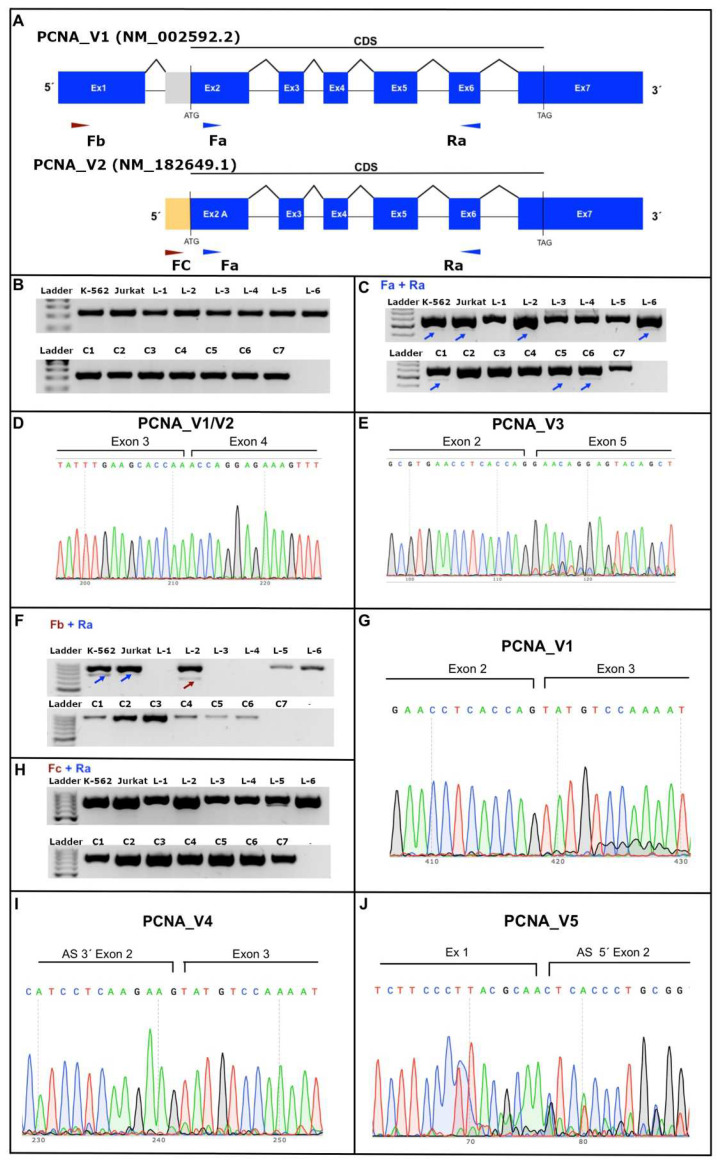
PCNA transcript variants expressed in healthy individuals, B-ALL patients and B-ALL cell lines. (**A**) The figure shows the primers designed to amplify the PCNA_V1 (NM_002592.2) and PCNA_V2 (NM_182649.1) primers. (**B**) The figure shows the housekeeping RPL4 expression in the cell lines (K562 and Jurkat), B-ALL patients (L1-L6) and healthy controls (C1-C7). (**C**) Amplification of the consensus sequence of PCNA transcript variants (NM_002592.2/NM_182649.1) using the primers Fa + Ra; note a PCR product of the ~600 bp PCNA canonical sequence, as expected. Additionally, we observed other PCR products of less size (~500 bp; blue arrows). (**D**) PCNA sequence of the PCR product of ~600 bp NM_002592.2 and/or NM_182649.1 (PCNA V1/V2) transcript variants, which show the boundary sites between exons 3 and 4. (**E**) PCNA_V3 sequence of an additional PCR product (~500 bp), showing an exon skipping of exons 3 and 4 of the PCNA mRNA transcript. (**F**) Amplification of the 5′End sequence of the PCNA transcript using Fb + Ra primers; note two additional PCR products at ~800 (blue arrow PCNA_V1, NM_002592.2) and ~700 bp (dark red arrow). (**G**) Representative sequence of PCNA transcript variant 1 (PCNA_V1, NM_002592.2), which shows the boundary site between exons 2 and 3. (**H**) Amplification of the 5′End sequence of the PCNA transcript, using Fc + Ra primers, the PCR products at ~800 (PCNA_V2, NM_182649.1). (**G**) Representative sequence of PCNA transcript variant 1 (PCNA_V1, NM_002592.2), which shows the boundary site between exons 2 and 3. (**I**) Representative sequence of novel PCNA transcript variant 4 (PCNA_V4) showing partial 5′ skipping in exon two. (**J**) Representative sequence of novel PCNA transcript variant 5 (PCNA_V5) showing partial 3′ skipping in exon two.

We then conducted mRNA sequencing to identify potential novel PCNA transcript variants, so we designed several pairs of primers, as shown in Table S1 and Figure 3A. First, we analyzed the expression of the housekeeping gene RPL4 in healthy controls, leukemia patients and leukemia cell lines (Figure 3B). We then amplified a fragment of both PCNA transcript variants (NM_002592.2/NM_182649.1) using the primers Fa + Ra, and we observed a PCR product of ~600 bp, as expected (Figure 3C). Nonetheless, we observed other PCR products of less size (~500 bp), which are shown with the blue arrow in Figure 3C. Both PCR products were purified and sequenced, and the canonical PCNA sequence (~600 bp) is shown in Figure 3D (NM_002592.2 and/or NM_182649.1). The additional PCR product (~500 bp) showed a cleavage in the sequence of exons 3 and 4 of the PCNA mRNA transcript (Figure 3E), so we named PCNA transcript variant 3 (PCNA_V3), which we consider to be a novel PCNA transcript variant because there is no information about its sequence in the National Center for Biotechnology Information (NCBI) database.

To determine the 5′End sequence of the PCNA transcript, we amplified using the combinatory primers Fb + Ra and Fc + Ra, as shown in Figure 3A. Suppressively, we observed two additional PCR products at ~800 (blue arrow) and ~700 bp (dark red arrow), as shown in Figure 3F. As expected, with our design Fb + Ra, we selectively amplified PCNA transcript variant 1 (PCNA_V1, NM_002592.2) (Figure 3G). The blue and dark red arrows show additional transcript variants expressed, which were denominated PCNA transcript variant 4 (PCNA_V4) and PCNA transcript variant 5 (PCNA_V5), and the sequences are shown in Figure 3I and 3J, respectively. PCNA_V4 showed partial 5′ skipping in exon two, and PCNA_V5 showed partial 3′ skipping in exon two. However, we could not obtain a clean sequence of PCNA_V5. For this reason, we excluded it from subsequent experiments.

**Figure 4:**
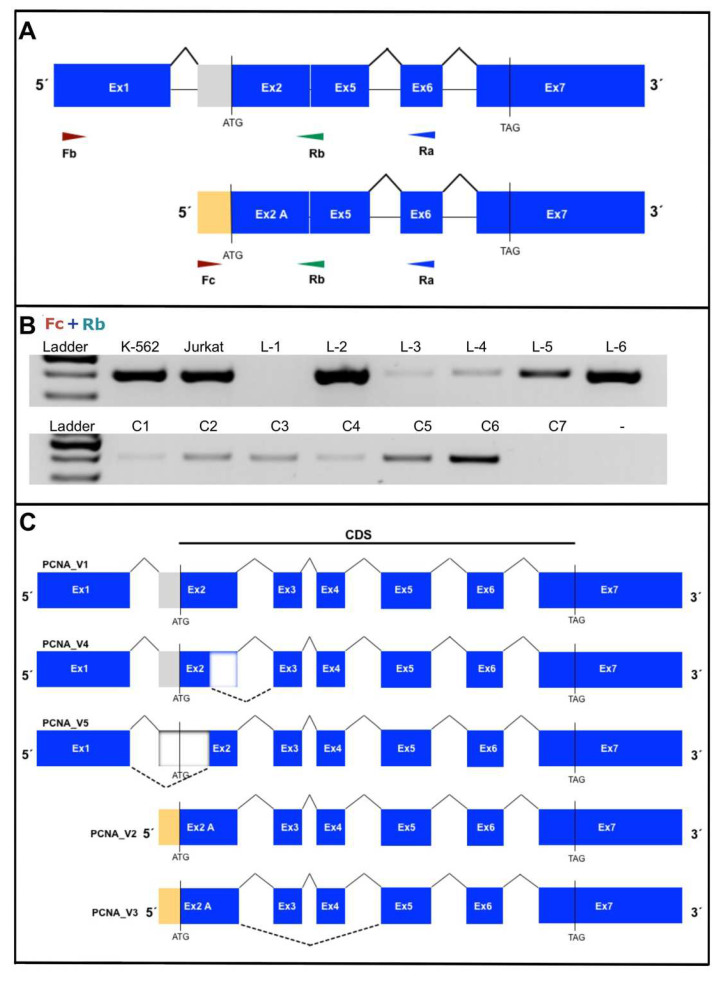
Amplification of the novel PCNA transcript variant 3 (PCNA_V3). (**A**) The figure shows the specific design of PCR primers to amplify PCNA_V3, which is located in the boundary site between exons 2 and 5. (**B**) Specific amplification of PCNA_V3 shows the PCR product at ~400 bp using Fc + Rb primers. (**C**) The figure shows the alternative splice model of the PCNA transcript variants expressed in B-ALL and healthy controls. Apparently, the transcript variants PCNA_V1, PCNA_V4 and PCNA_V5 follow the same splicing process and a different way for variants PCNA_V2 and PCNA_V3.

Subsequently, we amplified the PCNA transcript using the combinatory Fc + Ra primers (Figure 3H). Nevertheless, we observed high expression of PCNA transcript variant 2 (PCNA_V2), and it was not possible to observe the expression of PCNA_V3. Thus, we designed specific primers for amplification of PCNA_V3 (Figure 4A). We used the primers Fc + Rb, and the PCR showed only one PCR product, as expected (Figure 4B). However, we did not observe amplification with the primers Fb + Rb. Our analysis showed that the PCNA gene could express several transcript variants, as shown in Figure 4C.

### 3.2. Computational Analysis of the PCNA Transcript Variants

AS promotes changes in the mRNA sequence and consequently in the resulting protein. Thus, we used the ATGpr program [32] to perform in silico translation of the PCNA_V1, V2, V3, and V4 sequences to determine the hypothetical resulting proteins. As expected, PCNA_V1 and V2 are translated to the same protein of 261 amino acids. However, PCNA_V3 and V4 had reduced peptide sizes to 80 and 253 amino acids, respectively (Table S2).

We then performed a homology analysis using the PCNA sequence (GenBank: CAG46598.1) as a reference. Unsurprisingly, and according to the UNIPROT database [33] (entry P12004), our analysis showed several posttranslational modifications in PCNA_V3 and V4-resulting proteins (Table S3). Additionally, the aligned protein structure-based sequences showed several modifications in these proteins that are very important in the activity of the native PCNA protein. We think that PCNA_V3 would present changes in function because the predicted protein has missing domains that play central roles in protein transport and DNA binding, while PCNA_V4 has a shift in sequence, and these modifications do not promote losses in the structural domains (Table S4).

After that, we performed homology-based protein modeling using the Robetta server [21]. Our analysis showed high and good reliability using several templates. Moreover, the analysis of the dihedral angles φ and ψ (Ramachandran diagram) shows that the models have good quality since the amino acids have their dihedral angles mostly in the favored area. In the case of PCNA_V4, despite having a lower percentage of amino acids in the favored region, the server was able to provide a tertiary structure that is consistent with the structure of the native PCNA protein (Table S5, Figure S1).

**Figure 5:**
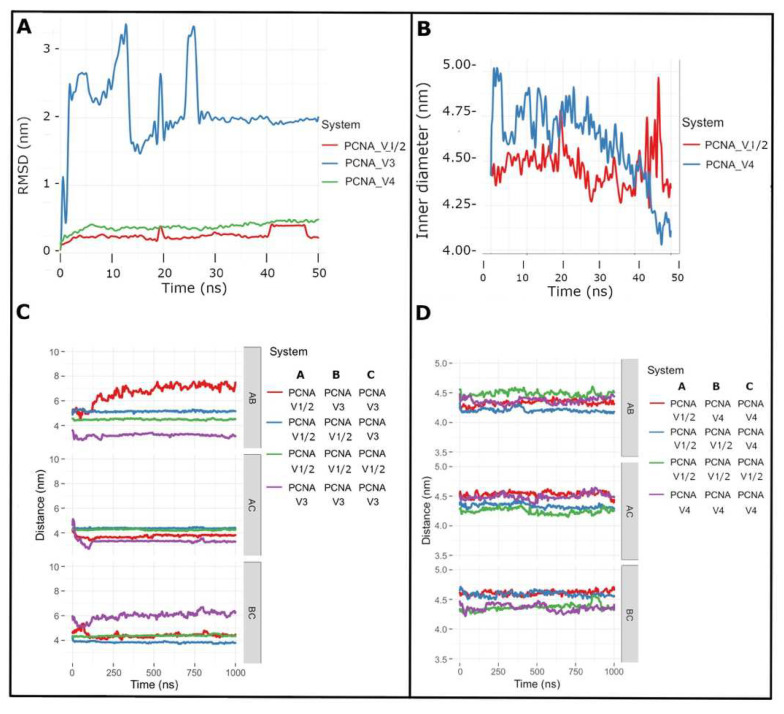
Computational analysis of different PCNA trimers. (**A**) PCNA protein (PCNA_V1/2) shows great structural stability in the system. The reduced PCNA_V3 resulted in a large conformational change in solution (blue line). Notice that PCNA_V4 (green line) is similar to the PCNA native structure. (**B**) PCNA_V4 presents a greater variability of the internal diameter of the homotrimer, which is relevant to the stability of the PCNA-DNA complex. (**C**) PCNA_V3 with native PCNA tends to form dimers with a highly variable structure (**A**,**B**, red line). Distances are shown in pairs: AB, AC and BC. Depending on the color, the distance of the analyzed pair is indicated; for example, the red line analyzes the heterotrimer formed by a native PCNA monomer (**A**) and a variant PCNA dimer (**B**,**C**), the same as for the blue line, the heterotrimer formed by a native PNCA dimer (**A**,**B**) and a variant PCNA monomer (**C**). The green and purple lines correspond to the homotrimers of native PCNA (green) or variant PCNA (purple). (**D**) PCNA_V4 and the native state protein tended to form stable homotrimers with a similar distance during the simulation time.

By aligning the complexes with respect to the native structure, several models were obtained; then, we performed molecular dynamics simulations to establish the behavior of trimers in solution (all-atom simulations, 50 ns). The structures obtained from the simulation of atomistic molecular dynamics were clustered according to the root-mean-square deviation (RMSD). First, we analyzed the native PCNA protein (PCNA_V1/V2, NM_002592.2/NM_182649.2), and we observed that PCNA showed great structural stability in the system with few changes, as shown in Figure 5A, presenting 6 representative structures that can interconvert their structure up to 16 transitions. As expected, the reduction in the size of PCNA_V3, because of the lack of a B domain, resulted in a large conformational change in solution (Figure 5A). This variant is not expected to have the functionality of the native structure. Moreover, PCNA_V4 was similar to the native structure (Figure 5A), presenting an IDCL with greater flexibility and without a constant secondary structure. The J loop was smaller and less similar to that of the native state, so it would be expected that the union with the polymerases would be diminished or even prevented (Figure S2). Additionally, PCNA_V4 presents less movement in its structure than native PCNA (Figure 5A), which would compromise the original function as a DNA polymerase cofactor, since PCNA_V4 would appear to be more rigid in a system that also requires some flexibility to mediate DNA binding for replication; finally, the DNA-binding site for PCNA_V4 presented a greater variability of the internal diameter of the PCNA-DNA complex (Figure 5B) which might be relevant to its stability. Nevertheless, these conformational changes could also suggest other types of functionalities, in addition to those of native PCNA, if these different conformations could be experimentally confirmed.

Similar to the atomistic simulations, the distances between the monomers in the study systems were measured, and the results are shown in Figure 5C,D. The results of the distances between monomers confirm the observations made in the systems in solution, showing that PCNA_V3 tends to form dimers whose structure is highly variable (Figure 5C). Moreover, the association of PCNA_V3 with PCNA-forming trimers shows significant variability (Figure 5C), so it might suggest that the interaction of PCNA_V3 with the back face of PCNA would be in a potentially detrimental balance for the intrinsic activity of the native structure. However, PCNA_V4 and the native state protein tended to form stable homotrimers with a constant distance during the simulation time (Figure 5D). Our results suggest that PCNA transcript variants 3 and 4 could have a significant function in cell processes.

### 3.3. The Molecular Dynamics Simulations Predicted Possible Interactions among the PCNA Transcript Variants

**Figure 6:**
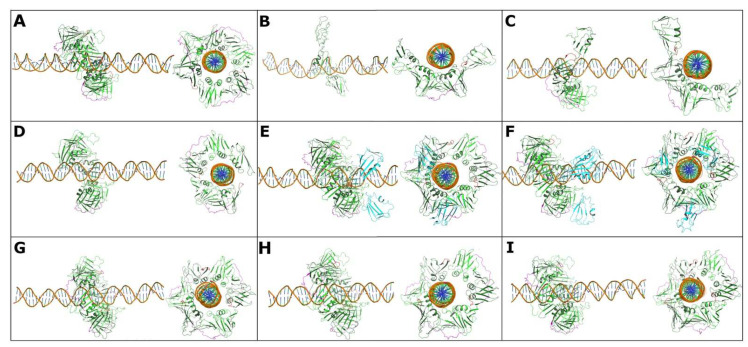
Representative conformations of different combinations of PCNA trimers. (**A**) PCNA native homotrimer showing a balanced structure where the DNA is located at the center of the homotrimer. (**B**) PCNA_V3 homodimer interacting with DNA. (**C**) Interaction of the PNCA_V3 homodimer with the PCNA native monomer and (**D**) the PCNA_V3 monomer with the PCNA native homodimer. (**E**) Interaction of PCNA native trimer (green) with PCNA_V3 homodimer (cyan), and (**F**) PCNA native homotrimer (green) with PCNA_V3 homotrimer (cyan). (**G**) PCNA_V4 homotrimer, also showing a relatively stable complex with DNA. (**H**) Interaction of the PCNA_V4 homodimer with the PCNA native monomer. (**I**) Interaction of the PCNA_V4 monomer with the PCNA native homodimer.

To determine the behavior of PCNA transcript variant-DNA complexes, we performed coarse-grained molecular dynamics simulations of 1 μs. In the case of the native PCNA, as expected, the most representative structure was at 551.14 ns, where the complex conformations have a balanced structure and the DNA is located exactly at the center of the homotrimer. Although not well appreciated, the structure maintains an angle close to 40° with respect to the DNA in a plane tilted toward the front face of PCNA, exposing important residues for polymerase recruitment (Figure 6A).

To observe the influence of PCNA_V3 expression on normal PCNA activity, systems were constructed where the native homotrimer was present with a different number of PCNA_V3 monomers. The DNA structure shows a considerable twist compared to when PCNA_V3 is not present, which would indicate that, despite forming a stable complex with PCNA-DNA, the reading frame could be affected by the presence of variants. On the other hand, PCNA_V3 monomers tend to preferentially interact with the rear face of PCNA, where residue K164 is found, which is very important for the regulation of PCNA activity by ubiquitination and SUMOylation. This would significantly affect the functionality of native PCNA. Alternatively, the PCNA_V3 trimer is unstable and disintegrates in the simulation time. Despite the influence of the negative surface of the DNA attracting the monomers, they are simply too small to form a stable trimer; however, PCNA_V3 forms stable dimers, as seen in their most representative structure at 530.60 ns, that do maintain interaction with DNA (Figure 6B). Additionally, the PCNA_V3 homodimer interacts with a PCNA_V1/V2 monomer, and the representative structure at 333.7 ns is shown in Figure 6C. The PCNA_V3 monomer with a PCNA_V1/V2 homodimer represented at 542.32 ns is shown in Figure 6D. The PCNA_V3 homodimer with a PCNA_V1/V2 homotrimer, stabilized early at 106.42 ns, is shown in Figure 6E, and the PCNA_V3 homotrimer with a PCNA_V1/V2 homotrimer, with a representative structure at 325.4 ns, is shown in Figure 6F.

Analogous to the native structure, the PCNA_V4 homotrimer forms a relatively stable complex with DNA at 618.02 ns (Figure 6G). However, it presents a considerable twist in all the structures where PCNA is present, which can significantly affect the reading frame. Likewise, the DNA tends not to be balanced in the center of the trimer, having a smaller distance from PCNA, which could be explained by the fact that the native structure has positively charged amino acids in the binding site. The most stable interaction of PCNA_V4 with PCNA_V1/2 is as follows: the PCNA_V4 homodimer interacts with a PCNA monomer at 810.24 ns (Figure 6H), and the PCNA_V4 monomer interacts with a homodimer of PCNA_V1/V2 at 884.46 ns (Figure 6I).

It is worth mentioning that PCNA has to be associated with replication factor C (RFC) and open to enter the DNA reading frame, so greater stability would not guarantee efficient binding with RFC, the formation of a functional complex or binding with DNA. In this sense, the global fluctuation per residue (RMSF) in Figure S2 shows that the native version has a greater fluctuation in the movement of its amino acids, which could enhance its association with RFC and, subsequently, with DNA.

### 3.4. Knockdown of the PCNA Transcript Variants V3 and V4 Promotes Cell Migration

To determine the malignant characteristics of PCNA V3 and V4 variants in vitro and identify a good model of knockdown, we analyzed the mRNA expression in 25 cancer cell lines by means of quantitative RT-PCR (Figure S3). As expected, the expression level of PCNA_V3 was less than that of PCNA_V4, and apparently, the amount of transcript variant expression was dependent on malignance. We identified HTR 8/SVneo as the cell line with the highest expression of PCNA_V3 and V4, so we used these cell lines for subsequent analysis.

**Figure 7:**
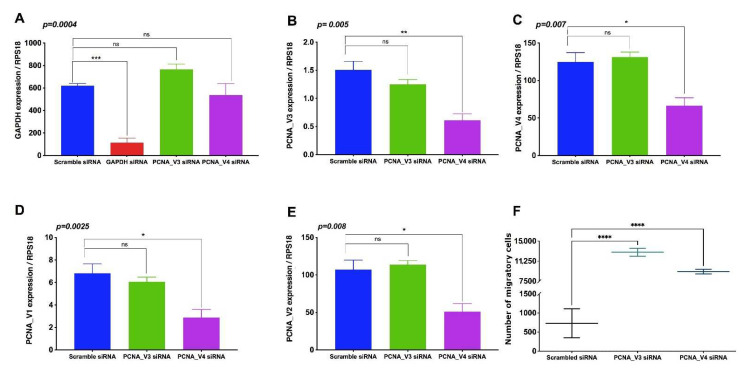
Bar graphs depict the mRNA silencing of the PCNA transcript variants and cells. The plot shows the absolute mRNA quantification using digital droplet RT-PCR. The interference model included scramble siRNA (blue box), positive control siRNA (GAPDH, red box), PCNA_V3 siRNA (green box) and PCNA_V4 siRNA (purple box). (**A**) The graph shows the quantification of GAPDH expression, and we observed significant silencing of the positive control GAPDH. We did not observe changes in expression in the scramble, PCNA_V3 and V4 siRNA groups. (**B**) Quantification of PCNA_V3 expression. The graph shows a 25% decrease in the level of expression vs. scramble; however, surprisingly, PCNA_V4 decreased its level of expression by ~60%. (**C**) Quantification of PCNA_V4 expression. The plot showed significant inhibition of PCNA_V4, as expected. However, in PCNA_V3, no changes in expression levels were observed. (**D**) Quantification of PCNA_V1 expression. We observed a subtle decrease in the level of PCNA_V3 siRNA expression and significant silencing by PCNA_V4 siRNA. (**E**) Quantification of PCNA_V2 expression. The plot showed significant silencing by PCNA_V4 siRNA; however, there were no differences by PCNA_V3 siRNA. (**F**) In the plot showing the transwell migration assay, we observed a significant increase in the number of migrating cells in the two siRNAs (PCNA_V3 and PCNA_V4). One-way ANOVA followed by Dunnett’s post-hoc test was considered statistically significant **** *p* < 0.0001, *** *p* < 0.001, ** *p* < 0.01, * *p* < 0.05, no significant (ns).

After cell transfection with siRNA PCNA_V3 and V4, we measured the mRNA inhibition by ddPCR of the PCNA transcript variants. We employed a positive control (GAPDH) and a negative control on-target siRNA. We observed significant silencing of the positive control (GAPDH), while in the cells treated with PCNA_V3 and V4 siRNA, we did not observe significant changes in their expression, as expected (Figure 7A). The inhibition of PCNA_V3 by the siRNA showed a tendency toward inhibition; however, the results were not significant (Figure 7B). Interestingly, we observed significantly lower levels of PCNA_V4 following the inhibition of PCNA_V3. This may be because PCNA_V3 is a regulator of PCNA_V4. On the other hand, we corroborated the significant inhibition of PCNA_V4 by siRNA, and we did not observe inhibition of PCNA_V3 (Figure 7C).

Additionally, we evaluated the expression of PCNA_V1 and V2 in the silencing model of PCNA_V3 and V4. In the PCNA_V3 siRNA model, we observed less PCNA_V1 but not PCNA_V2. However, the data were not significant (Figure 7D,E). In the PCNA_V4 siRNA model, we observed that both variants significantly decreased their expression (Figure 7D,E). After that, we analyzed cell proliferation by means of MMT assay. We observed a tendency to increase cell proliferation in the cells that were inhibited with PCNA_V4. However, the data were not significant. Additionally, we consider that HTR8/SVneo cells have hyperinvasiveness capacities, so we measured the impact of cell migration in the PCNA_V3 and V4 siRNA models. Surprisingly, the inhibition of the PCNA transcript variants promoted the significant cell migration of HTR8/SVneo cells (Figure 7F).

### 3.5. Proliferating Cell Nuclear Antigen Transcript Variants Are Overexpressed in B-ALL, and Low Expression Is Associated with Relapse

**Figure 8:**
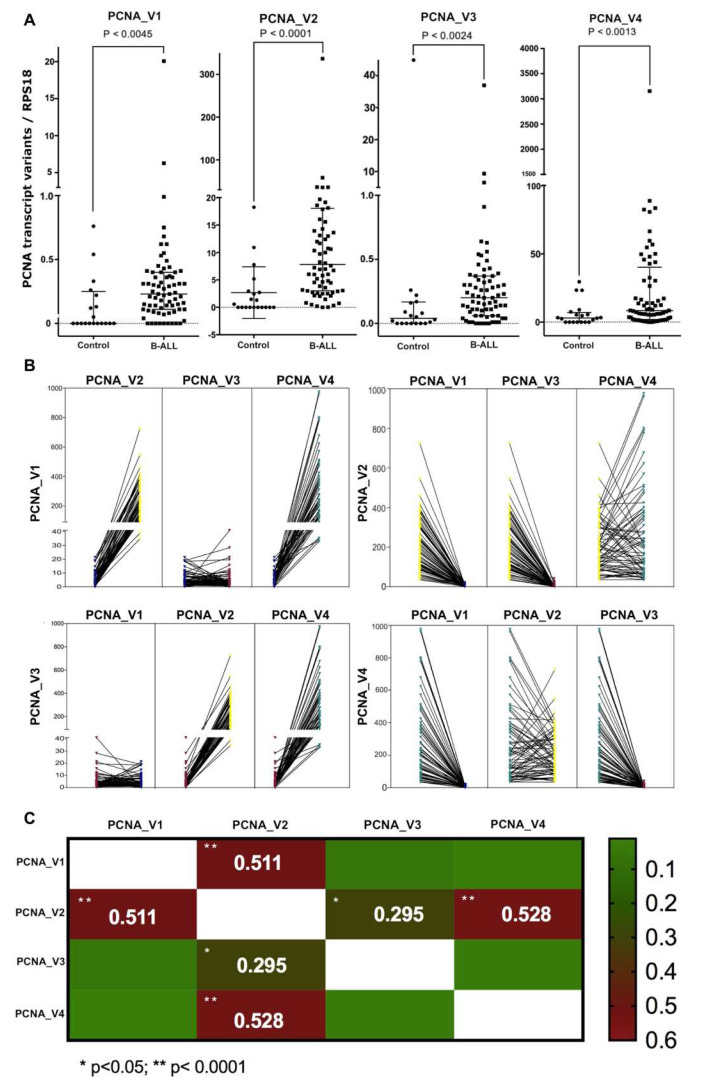
Expression of PCNA transcript variants in B-cell ALL patients. We performed absolute quantification using digital droplet RT-PCR. The *y*-axis shows the mRNA copy number of the PCNA transcript variants. The *x*-axis shows the healthy peripheral blood controls and B-ALL patients. (**A**) We observed significant overexpression of the PCNA transcript variants in B-LL patients. The four transcripts showed different levels of expression; the least expressed was PCNA_V1, followed by PCNA_V3 and PCNA_V2, and the most expressed was PCNA_V4 (Mann-Whitney U test). (**B**) The plot shows the comparison of the expression levels of the four PCNA transcript variants in all B-ALL patients analyzed (Mann-Whitney U test). (**C**) The heatmap shows Pearson’s correlation. Our results showed a significant correlation between PCNA_V1 and PCNA_V2, PCNA_V2 and PCNA_V3, and PCNA_V2 and PCNA_V4.

To determine the level of expression of each PCNA transcript variant, we performed gene expression quantification using digital droplet RT-PCR, and the PCR amplification plot is shown in Figure S4. Then, we compared the absolute gene expression of the RPS18 housekeeping gene against each PCNA transcript variant, and we compared B-ALL patients against healthy peripheral blood controls. The clinical characteristics of the B-ALL patients are shown in the Table S6. The analysis showed that all transcript variants showed differential expression among them. The least expressed was PCNA_V1, followed by PCNA_V3 and PCNA_V2, and the most expressed was PCNA_V4. PCNA_V1 and V3 maintained the same low expression pattern (Figure 8A).

We found that the expression of all PCNA transcript variants was weak in the healthy controls, but in the B-ALL patients, they were overexpressed (Figure 8A). These results indicate that PCNA_V1, V2, V3 and V4 are upregulated in B-ALL. Based on the structure sequence and their expression in B-ALL, we think that the transcript variants could share patterns of the regulation expression. However, Pearson’s correlation showed that only PCNA_V2 expression correlated with all PCNA transcript variants (Figure 8B,C). These results suggest that there is some regulatory mechanism between the transcript variants of PCNA.

**Figure 9:**
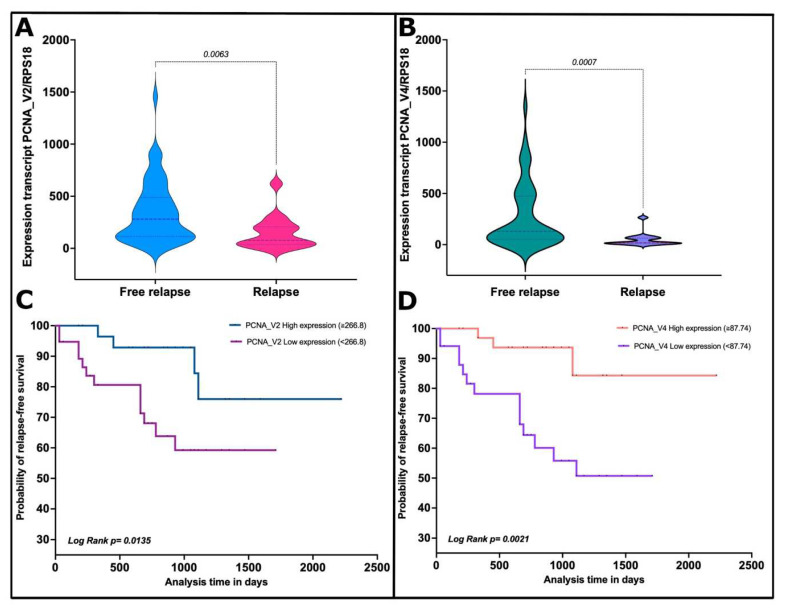
PCNA_V2 and PCNA_V4 transcript variant expression is a potential predictor of relapse in B-ALL. The plot shows the absolute gene expression of the PCNA transcript variants normalized vs. housekeeping RPS18 transcript. (**A**) The *y*-axis shows the expression levels of PCNA_V2. The *x*-axis shows the patient groups: blue = free relapse patients and pink = relapse patients. The relapsed patients showed a significant reduction in PCNA_V2 expression vs. free relapse (*p* value = 0.0063; Mann-Whitney U test). (**B**) The *y*-axis shows the expression levels of PCNA_V4. The *x*-axis shows the patient groups: green = free relapse patients and purple = relapse patients. The relapsed patients showed a significant reduction in PCNA_V4 expression vs. free relapse (*p* value = 0.0007; Mann-Whitney U test). (**C**,**D**) The plots show the Kaplan-Meier curves for relapse-free survival based on PCNA_V2 and PCNA_V4 expression levels. The *y*-axis shows the relapse-free survival probability, and the *x*-axis shows the time in days. The plots showed that the low expression of PCNA_2 (266.8 PCNA_V2/RPS18) and V4 (87.74 PCNA_V4/RPS18) in B-ALL patients increased the risk of relapse (*p* value = 0.0135 and 0.0021, respectively).

We thus categorized the patients based on clinical features, and we observed that PCNA_V2 and V4 were significantly suppressed in the patents with relapse (*p* values = 0.0063 and 0.0007, Figure 9A,B). Additionally, we generated an ROC curve to determine the power of classification based on relapse. PCNA_V2 showed an AUC = 0.729 (CI 95%, 0.587–0.871) *p* value = 0.007, Youden Index = 0.386, sensitivity = 86.6, specificity = 51.9, and PCNA_V4 showed an AUC = 0.780 (CI 95%, 0.664–0.896) *p* value = 0.001, Youden Index = 0.549, sensitivity = 93.3, specificity = 61.5. We then performed a Kaplan-Meier free relapse curve to estimate the free relapse survival and determine the potential prognosis of PCNA_V2 and V4 (Figure 9C,D). We found that high expression of PCNA_V2 and V4 is associated with overall relapse-free survival in B-ALL. Moreover, high PCNA_V2 and V4 expression was associated with significantly improved relapse-free survival compared with low PCNA_V2 and V4 expression.

In addition, data were divided based on the expression levels of PCNA_V2 and V4 with the cutoff value set with the ROC curve analysis (PCNA_V2, <266.8; PCNA_V4, <87.74). When median or relapse-free survival (RFS) was analyzed in patients with high and low PCNA_V2 expression, low PCNA_V2 expression was associated with a shorter time to relapse (*p* value = 0.0135; HR, 3.14; 95% CI, 1.21–8.13) (Figure 9C). For PCNA_V4 expression, low PCNA_V4 expression was also associated with a shorter time to relapse (*p* = 0.0021; HR, 5.3; 95% CI, 2–13.8) (Figure 9D).

## 4. Discussion

Leukemia is the most common childhood malignancy worldwide, and its risk classification is well known, as are the molecular DNA alterations associated with it [34,35,36,37,38]. AS is a very important posttranscriptional process that increases the mRNA variety expressed and consequently the protein diversity [39,40,41]. Some studies have shown that up to 95% of genes can be processed by AS [42,43]. In cancer, AS is deregulated, so this event promotes the expression of atypical transcript variants, which are known to contribute to the malignance phenotype [6,7,8]. However, the whole transcriptome expressed in cancer cells and the molecular prognostic factors associated with adverse clinical events are not completely understood.

In this context, since PCNA is overexpressed in several types of cancer, but the diversity of its transcript variants in this condition is unknown, we characterized the expression, structure and implications as predictors of relapse of some novel PCNA transcript variants. Our results obtained by means of microarrays, RT-PCR, and sequencing showed three additional transcripts, i.e., PCNA_V3, PCNA_V4, and PCNA_V5, overexpressed in B-ALL cell lines and B-ALL patients. Because we did not obtain a completely clean sequence of the PCNA_V5 transcript, we excluded it from subsequent experiments. Additionally, our findings showed that the new PCNA transcript variants were expressed (but not overexpressed) in healthy controls, suggesting that their expression is not exclusively of the B-ALL disease. Although the comparison between peripheral blood and bone marrow is not the best due to cellular composition, it is the best approach we have to demonstrate the increased expression of PCNA variants.

For several years, it was very well known that the PCNA gene can give rise to two mRNA transcripts (NM_002592.2, PCNA_V1 and NM_182649.2, PCNA_V2) that code for the same protein [44]. However, until now, there has been no evidence of other transcript variants of PCNA expressed in healthy individuals or in cancer disease. The PCNA protein is a master protein because it acts as scaffolding in the interaction with DNA and with a great diversity of regulator proteins, which play an important function in cell biology, such as cell replication, clamp loader, DNA ligase, mismatch repair, ubiquitination, base excision repair, nucleotide excision repair, chromatin remodeling, histone acetylation, DNA methylation, histone deacetylation, cell cycle, and apoptosis [11]. Additionally, PCNA plays an important role in cancer because it is overexpressed in multiple cancer types, including non-small cell lung cancer [45], hepatocellular carcinoma [46], breast cancer [47], and pancreatic cancer [48], as well as in our present B-ALL results, suggesting that PCNA overexpression is a potential marker for many cancer types.

Our in-silico approach showed that the new PCNA transcript variants could be translated to proteins [32,49,50,51]. In addition, our analysis displayed several modifications in the resulting PCNA_V3 and PCNA_V4 transcript variants. The most substantial modification was observed in PCNA_V3 by the reduction of the B domain and loss of the DNA-binding site and Loop J, which are essential in native PCNA function because they recognize several proteins that are indispensable in DNA repair, DNA polymerase, DNA methylation, and DNA ligation, among others [52,53,54,55,56]. These results suggest that the novel variant could play other functions. Moreover, PCNA_V4 does not lose the essential PCNA domains. However, we think that several subtle changes could contribute to topological reordinations in the resulting protein, consequently modifying the formation of the homotrimer protein and thus changing its function.

Additionally, we showed that native PCNA could interact with PCNA_V3 and PCNA_V4 on a long-time scale [57]. PCNA_V3 presented an inability to form stable trimers but may form substructures that interact with the DNA on its positive side. However, DNA preferentially interacted with PCNA than with the PCNA_V3 variant, which could mean that the absent part of domain II, in this variant is also important for interaction with DNA, albeit indirectly. The association of PCNA_V3 with PCNA-forming trimers and their significant variability suggest that the interaction of PCNA_V3 with the back face of PCNA would be in a potentially detrimental balance for the intrinsic activity of the native structure. Moreover, the PCNAV_4 variant and the native state protein tended to form stable homotrimers with a constant distance during the simulation time. However, it presented a considerable twist in all the structures where PCNA was present, which may affect the reading frame and their interaction with DNA. In brief, the interaction of these variants could lead to a wider diversity of functions in the biology of the cell, such as an important role in DNA repair and apoptosis, as well as in cancer cells [11].

On the other hand, several studies have shown that aberrant transcript variants contribute to malignant processes, such as TP53 [58], PTB [59], BIM, BIN, [60], SRSF6 [61], and PRPF6 [62]. However, our results showed that the PCNA transcript variants are not aberrant transcripts because we found their expression in healthy controls. Additionally, the inhibition of PCNA_V3 and PCNA_V4 increased migration in vitro. This result suggests that these transcript variants play protective actions in the cell and that a thin downregulation could exacerbate the malignance process. Moreover, all PCNA transcript variants were overexpressed in B-ALL cells; thus, our results are very interesting because for the first time, we identified alternative PCNA transcripts and quantified them, showing the same high expression of canonical PCNA in non-small cell lung cancer [45] and oral squamous cell carcinoma [63], among others. Since we observed the overexpression of canonical PCNA in B-ALL, it could be that the PCNA transcript variants are also overexpressed in other cancer types.

Finally, Leif E. Peterson and Tatiana Kovyrshina performed metagene analysis and observed that PCNA predicted that overall survival mediates DNA repair adjustment in diverse cancer types, including breast, cervical, colorectal, liver, lung, small cell lung, ovarian, melanoma, renal, stomach, and uterine cancer [64]. In addition, Guo, J. L et al. observed the overexpression of PCNA in a series of cases of breast cancer [65] and Dan-Dan, Li et al. observed it in hepatocellular carcinoma [46]. Additionally, Dan-Dan, Li et al. correlated the expression level with clinicopathological stage. Our results showed that PCNA is overexpressed in B-ALL cells vs. healthy peripheral blood controls and when we analyzed the clinicopathological features, we observed a significant reduction in the expression of PCNA_V4 and PCNA_V2, and this low expression correlated with relapse. Moreover, the in vitro silencing of PCNA_V3 and V4 exacerbated the migration capacities of HTR8/SVneo cells, suggesting protective actions of these PCNA variants. However, it will be necessary to carry out subsequent assays in a leukemia model. Since the in vitro results were in agreement with the clinicopathological features and low expression of PCNA_V2 and PCNA_V4 was observed in relapsed patients, our results suggest that these transcript variants are potential molecular markers of relapse in acute lymphoblastic leukemia patients.

## 5. Conclusions

In this work, for the first time, we identified new PCNA transcript variants that were apparently overexpressed in B-ALL cells. The bioinformatic approach suggested that these PCNA transcript variants could interact with native PCNA, which may modify its functionality. Additionally, inhibition of these variants contributes to malignant characteristics in vitro. Moreover, PCNA_V2 and V4 could be used as potential molecular markers to predict relapse in leukemia patients. PCNA transcript variants were also found in healthy controls (with lower expression). It would be necessary to continue studying the diversity and functionality of the PCNA transcript variants expressed in healthy individuals and cancer patients.

## Figures and Tables

**Table 1:** Database used in data mining.

Tissue	GEO Accession
B-ALL	GSM1180810, GSM1180813, GSM1180787, GSM1180791, GSM1180795, GSM1180798, GSM1180801, GSM1180804, GSM1180807, GSM1180816
B-Cell	GSM1180818, GSM1180829, GSM1180841, GSM1180845
B- CL	GSM1180775 (KASUMI-2), GSM1180782 (KASUMI-2), GSM1180842 (KOPN-8), GSM1180846 (KOPN-8), GSM1180817 (MHH-CALL-4), GSM1180823 (MHH-CALL-4), GSM1180808 (MUTZ-5), GSM1180811 (MUTZ-5), GSM1180779 (NALM-6), GSM1180763 (NALM-6), GSM1180764 (SUP-B15), GSM1180780 (SUP-B15)

## Data Availability

Personal data of patients are not available for ethical reasons.

## Supplementary Materials

The following supporting information can be downloaded at: https://www.mdpi.com/article/10.3390/cells11203205/s1, Figure S1: Ramachandran plot and ribbon representation of PCNA_V3 and PCNA_V4 trimers. Models have good quality since the amino acids have their dihedral angles mostly in the favored area, Figure S2: Root-mean standard fluctuation (RMSF) of amino acids from relevant domains of the PCNA and PCNA_V4 trimers calculated by coarse-grained molecular dynamics simulations. Native PCNA has a greater fluctuation in the movement of its amino acids, which could change its association with DNA. Notice that PCNA_V4 presents an IDCL with greater flexibility and without a constant secondary structure; the J loop was smaller and less similar to that of the native state, Figure S3: Relative RT-PCR quantification of PCNA_V3 and PCNA_V4 expression in 26 cancer cell lines. The y-axis shows the 26 cancer cell lines, and the x-axis shows the relative gene expression 2^−∆∆Ct^ method [66]. The level of expression was compared vs. the leukemia cell line (SUP-B15). (A) The plot indicates the PCNA_V3 expression level. (B) The plot indicates PCNA_V4 expression. Note that the HTR 8/SVneo cell line presented the highest expression of PCNA_V3 and V4 transcript variants; *p* ˂ 0.0001, one-way ANOVA followed by Dunnett’s post-hoc test, Figure S4: Droplet digital PCR amplification of PCNA_V1, PCNA_V2, PCNA_V3 and PCNA_V4. The figure shows the representative amplification of the three B_ALL patients (ALL4, ALL5, ALL6) and one no-target template (NTC). The blue droplet represents a positive amplification, and the gray droplet represents a negative amplification. The pink line represents the threshold between positive and negative droplets, Table S1: Pairs of primers to amplify PCNA transcript variants, Table S2: In silico translation of the PCNA_V1, V2, V3, and V4 sequences, Table S3: Potential posttranslational modifications of PCNA_V3 and PCNA_V4. Table S4. Domains found in the new PCNA transcript variants, Table S5: Homology analysis of the novel PCNA transcript variants, Table S6: Clinical characteristics of the patient cohort.
