# Supplementary material for: The Proliferating Cell Nuclear Antigen (PCNA) Transcript Variants as Potential Relapse Markers in B-Cell Acute Lymphoblastic Leukemia
Source: Cells. 2022 Oct 12;11(20):3205. doi: 10.3390/cells11203205 (PMC9600710; doi:10.3390/cells11203205)
Supplement: Supplementary file 1 [file cells-11-03205-s001.zip › Table S1.pdf]

cells

MDPI

| Transcript | Primer sequence | TM | Amplicon size |
| --- | --- | --- | --- |
| **A. RT-PCR and sequencing transcript variants** | | | |
| **RPL4** | Forward: 5′ CGAATGAGAGCTGGCAAAGGCAAA 3′<br>Reverse: 5′ ACGCCAAGTGCCGTACAATTCATC 3′ | 60°C | 243 bp |
| **PCNA (Fa + Ra)** | Forward: 5′ ATATTAGCTCCAGCGGTGTAAA 3′<br>Reverse: 5′ ACATCTGCAGACATACTGAGTG 3′ | 55°C | 613 bp |
| **PCNA1** | Forward: 5′TGGTTCTGGAGGTAACCGG 3′<br>Reverse: 5′ ACATCTGCAGACATACTGAGTG 3′ | 55.5°C | 916 bp |
| **PCNA2** | Forward: 5′ ATTAAACGGTTGCAGGCGTAG 3′<br>Reverse: 5′ ACATCTGCAGACATACTGAGTG 3′ | 55.5°C | 858 bp |
| **PCNA3** | Forward: 5′ ATTAAACGGTTGCAGGCGTAG 3′<br>Reverse: 5′ TACTCCTGTTCCTGGTGAGGTT 3′ | 54.5°C | 425 bp |
| **B. 3´RACE System for Rapid Amplification of cDNA Ends RT-PCR** | | | |
| **3´RACE PCNA3** | Forward: 5′ AACCTCACCAGGAACAGGAGTAC 3′<br>Reverse: 5′ GGCCACGCGTCGACTAGTAC 3′ | 60.5°C | 740 bp |
| **3´RACE PCNA3** | Forward: 5′ GCTGTGTAGTAAAGATGCCTTCTG 3′<br>Reverse: 5′ GGCCACGCGTCGACTAGTAC 3′ | 60°C | 726 bp |

| | | | |
|---|---|---|---|
| **nested** | | | |
| **3′RACE PCNA4** | Forward: 5′GCTCCATCCTCAAGAAGTATGTCCAA 3′<br>Reverse: 5′ GGCCACGCGTCGACTAGTAC 3′ | 60.5°C | 912 bp |
| **3′RACE PCNA4 nested** | Forward: 5′ ATACTAAAATGCGCCGGCAATGAA 3′<br>Reverse: 5′ GGCCACGCGTCGACTAGTAC 3′ | 60°C | 885 bp |
| **C. Digital dropet RT-PCR expression of transcript variants** | | | |
| **RPS18** | Forward: 5′ CAGCCAGGTCCTAGCCAATG 3′<br>Reverse: 5′ CCCTCTATGGGCCCGAATCT 3′ | 60°C | 82 bp |
| **PCNA-1q** | Forward: 5′ CTGGTTCTGGAGGTAACCGGT 3′<br>Reverse: 5′ GACCGGCTGAGACTTGCGTAA 3′ | 60°C | 116 |
| **PCNA-2q** | Forward: 5′ CATTAAACGGTTGCAGGCGTAGC 3′<br>Reverse: 5′ CAACAACGCCGCTACAGGCA 3′ | 60°C | 148 bp |
| **PCNA-3q** | Forward: 5′ GGGATATTAGCTCCAGCGGTGT 3′<br>Reverse: 5′ GTACTCCTGTTCCTGGTGAGGTT 3′ | 60°C | 151 bp |
| **PCNA-4q** | Forward: 5′ TAGACTTTCCTCCTTCCCGCCTG 3′<br>Reverse: 5′ TTGGACATACTTCTTGAGGATGGAGC 3′ | 60°C | 105 bp |
