# Supplementary material for: The Proliferating Cell Nuclear Antigen (PCNA) Transcript Variants as Potential Relapse Markers in B-Cell Acute Lymphoblastic Leukemia
Source: Cells. 2022 Oct 12;11(20):3205. doi: 10.3390/cells11203205 (PMC9600710; doi:10.3390/cells11203205)
Supplement: Supplementary file 1 [file cells-11-03205-s001.zip › Table S2.pdf]

Transcript	No of ATG from 5´end	Relia-bility	Frame	Identity Kozak rule A/GXX-ATGG	Start (bp)	Finish (bp)	ORF Length (aa)	Stop codon found	Sequence
PCNA_V1	3	0.90	3	AXXATGt	240	1022	261	Yes	MFEARLVQGSILKKVLEALKDLINEACWDISSSGVNLQSMDSSH-VSLVQLT-LRSEGFDTYRCDRNLAMGVNLTSMSKILKCAGNEDIITLRAEDNADTLALVFEAPNQEKVSDYEMKLMDLDVEQLGI-PEQEYSCVVKMPSGEFARICRDLSHIGDAVVIS-CAKDGVKFSASGELGNGNIKLSQTSNVDKEEEAVTIEMNEPVQLTFALRYLNFFTKATPLSSTVTLSMSADVPLVVEYKIADMGHLKYYLAPKIEDEEGS
PCNA_V2	1	0.90	2	AXXATGt	160	943	261	Yes	MFEARLVQGSILKKVLEALKDLINEACWDISSSGVNLQSMDSSH-VSLVQLT-LRSEGFDTYRCDRNLAMGVNLTSMSKILKCAGNEDIITLRAEDNADTLALVFEAPNQEKVSDYEMKLMDLDVEQLGI-PEQEYSCVVKMPSGEFARICRDLSHIGDAVVIS-CAKDGVKFSASGELGNGNIKLSQTSNVDKEEEAVTIEMNEPVQLTFALRYLNFFTKATPLSSTVTLSMSADVPLVVEYKIADMGHLKYYLAPKIEDEEGS
PCNA_V3	1	0.86	3	AXXATGt	204	443	80	Yes	MFEARLVQGSILKKVLEALKDLINEACWDISSSGVNLQSMDSSH-VSLVQLTLRSEGFDTYRCDRNLAMGVNLTRNRSTAV
PCNA_V4	2	0.39	1	cXXATGc	85	843	253	Yes	MLADLGQSSSLTQVSAGRRDVRPLALRLLKPKPARLSSFP-PACSGVVATPPPCSRRAWS-RAPSSRSMSKILKCAGNEDIITLRAEDNADTLALVFEAPNQEKVSDYEMKLMDLDVEQLGIPEQEYSCVVKMPSGEFARICRDLSHIGDAVVIS-CAKDGVKFSASGELGNGNI-KLSQTSNVDKEEEAVTIEMNEPVQLTFALRYLNFFTKATPLSSTVTLSMSADVPLVVEYKIADMGHLKYYLAPKIEDEEGS
