# Supplementary material for: The Proliferating Cell Nuclear Antigen (PCNA) Transcript Variants as Potential Relapse Markers in B-Cell Acute Lymphoblastic Leukemia
Source: Cells. 2022 Oct 12;11(20):3205. doi: 10.3390/cells11203205 (PMC9600710; doi:10.3390/cells11203205)
Supplement: Supplementary file 1 [file cells-11-03205-s001.zip › Table S3, S4 and S5.pdf]

Table S3. Potential posttranslational modifications of PCNA_V3 and PCNA_V4.

Site modification	Description	PCNA_V3	PCNA_V4
**14**	N^6-Acety-L-lysine	Lys14	Gln13
**77**	N^6-Acety-L-lysine	-	Lys77
**80**	N^6-Acety-L-lysine	-	Lys80
**164**	Isopeptide glycillisin (Lys-Gly) (binding with G-Cter on SUMO2) (binding with G-Cter in ubiquitin)	-	Lys164
**211**	Phosphotyrosine	-	Tyr211
**248**	N^6-Acety-L-lysine	-	Lys240
**254**	Isopeptide glycillisin (Lys-Gly) (binding with G-Cter on SUMO2)	-	Lys246

Table S4. Domains found in the new PCNA transcript variants.

Domain	Site	PCNA_V3	PCNA_V4
**Domain I**	1-123	1-49, 88-94, 103-105	1-55, 63-123
**Domain II**	132-258	133-143, 180-186, 228-231	132-261
**IDCL**	118-134	133-134	118-134
**DNA-binding site**	60-80	-	63-80
**Loop J, Asp113, Gly178**	105-110, 113, 178	-	105-110, 113, 178

Table S5. Homology analysis of the novel PCNA transcript variants.

Model	Robetta		Ramachandran (PROCHECK[24])		
	**Aling reliability**	**Tamplete structure (PDB)**	**Favored**	**Allowed**	**No Allowed**
PCNA_V3	0.9771	PCNA *A. thaliana* (2ZVW)	85.1%	14.4%	0.5%
PCNA_V4	0.7951	PCNA *S. cerevisiae* (3K4X)	87.5%	12.5%	0.0%
