# Supplementary material for: The Proliferating Cell Nuclear Antigen (PCNA) Transcript Variants as Potential Relapse Markers in B-Cell Acute Lymphoblastic Leukemia
Source: Cells. 2022 Oct 12;11(20):3205. doi: 10.3390/cells11203205 (PMC9600710; doi:10.3390/cells11203205)
Supplement: Supplementary file 1 [file cells-11-03205-s001.zip › Table S6.pdf]

cells MDPI

Table S6. Clinical characteristics of the patient cohort.

Variable	Total %	PCNA_V2 expression	*p value*	PCNA_V4 expression	*p value*
**Age**					
**0-9**	40 (57.14%)	13 (18.5%) High<br>27 (38.5.%) Low	0.400	17 24.2%) High<br>23 (23.8%) Low	0.240
**>9**	30 (42.85%)	7 (10%) High<br>23 (32.8%) Low		17 (24.2%) High<br>13 (18.5%) Low	
**Sex**					
**Men**	34 (48.6%)	12 (17.1%) High<br>22 (31.4%) Low	0.347	27 (38.6) High<br>7 (10.0%) Low	0.094
**Women**	36 (51.4%)	9 (12.9%) High<br>27 (38.6%) Low		22 (31.4%) High<br>14 (20.0%) Low	
**Genetic findings: T(9;22), T(1;19), T(12;21)**					
**Yes**	7 (10%)	3 (4.2%) High<br>4 (5.7%) Low	0.433	3 (4.2%) High<br>4 (5.7.%) Low	0.433
**Cerebrospinal fluid (blasts %)**					
**No blast**	64 (91.4%)	19 (27.1%) High<br>45 (64.3%) Low	0.444	45 (64.3%) High<br>19 (27.1%) Low	0.444
**< 5 blast**	2 (2.9%%)	0 (0%) High<br>2 (2.9%) Low		2 (2.9%) High<br>0 (0%) Low	
**> 5 blast**	4 (5.7%)	2 (2.9%) High<br>2 (2.9%) Low		2 (2.9%) High<br>2 (2.9%) Low	
**Treatment response**					
**Good**	45 (64.3%)	16 (22.9%) High<br>29 (41.4%) Low	0.243	32 (45.7%) High<br>13 (18.6%) Low	0.894
**Bad**	23 (32.9%)	5 (7.1%) High<br>18 (25.7%) Low		16 (22.9%) High<br>7 (10%) Low	
**Hypodiploidy**					
**Yes**	23 (32.9%)	7 (10%) High<br>16 (22.9%) Low	0.955	15 (21.4%) High<br>8 (11.4%) Low	0.541
**No**	47 (67.1%)	14 (20.0%) High<br>33 (47.1%) Low		34 (48.6%) High<br>13 (16.6%) Low	
**Blast %**					
**<50**	33 (47.1%)	9 (12.9%) High<br>24 (34.3%) Low	0.638	20 (28.6%) High<br>13 (18.6%) Low	0.105
**>50**	37 (52.9%)	12 (17.1%) High<br>25 (35.7%) Low		29 (41.4%) High<br>8 (11.4%) Low	
**Relapse**					
**Yes**	22 (31.4%)	4 (5.7%) High<br>18 (25.7%) Low	0.118	10 (14.3%) High<br>12 (17.1%) Low	0.002
**No**	48 (68.6%)	17 (24.3%) High<br>31 (44.3%) Low		39 (55.7%) High<br>9 (12.9%) Low	
**Status**					
**Alive**	59 (84.3%)	19 (27.1%) High<br>40 (57.1%) Low	0.351	43 (61.4%) High<br>16 (22.9%) Low	0.223
**Died**	11 (15.7%)	2 (2.9%) High<br>9 (12.9%) Low		6 (8.6%) High<br>5 (7.1%) Low	
