# Supplementary figures and images for: The Proliferating Cell Nuclear Antigen (PCNA) Transcript Variants as Potential Relapse Markers in B-Cell Acute Lymphoblastic Leukemia
Source: Cells. 2022 Oct 12;11(20):3205. doi: 10.3390/cells11203205 (PMC9600710; doi:10.3390/cells11203205)

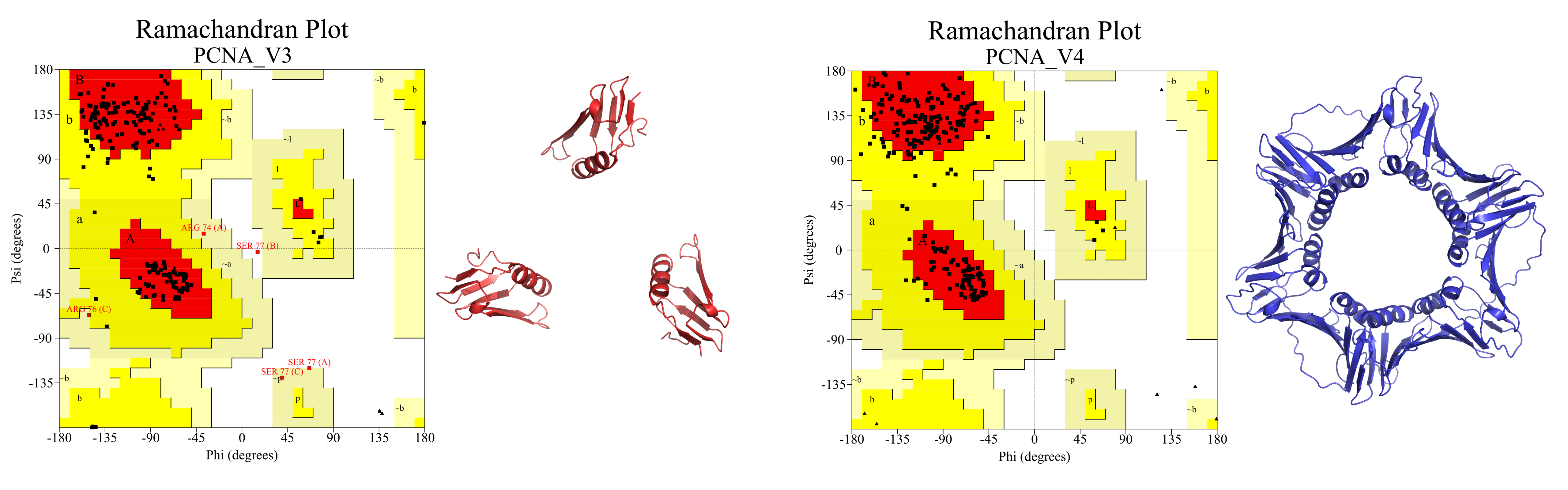

Supplement: Supplementary file 1 [file cells-11-03205-s001.zip › Figure S1.tiff]

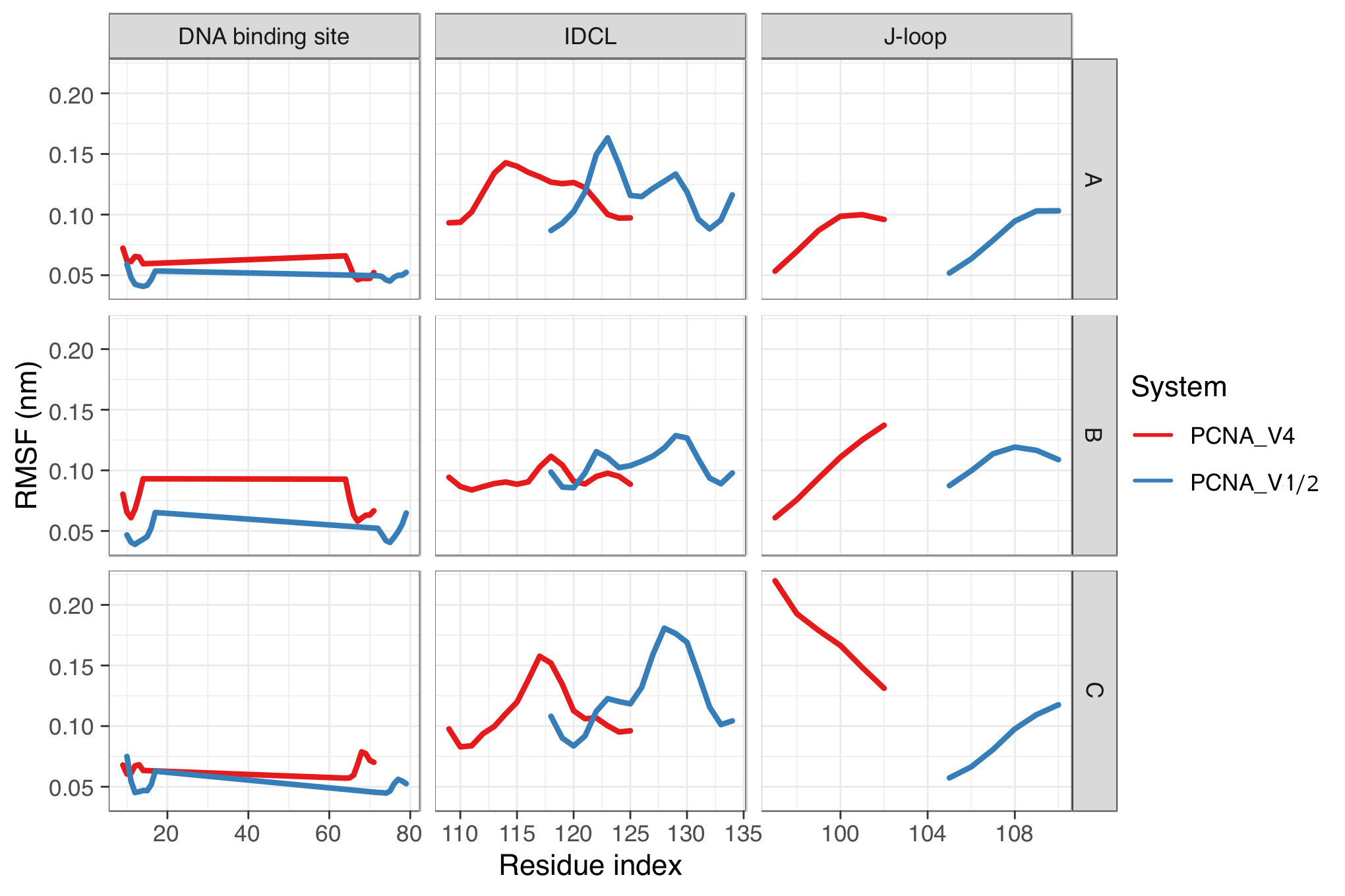

Supplement: Supplementary file 1 [file cells-11-03205-s001.zip › Figure S2.jpg]

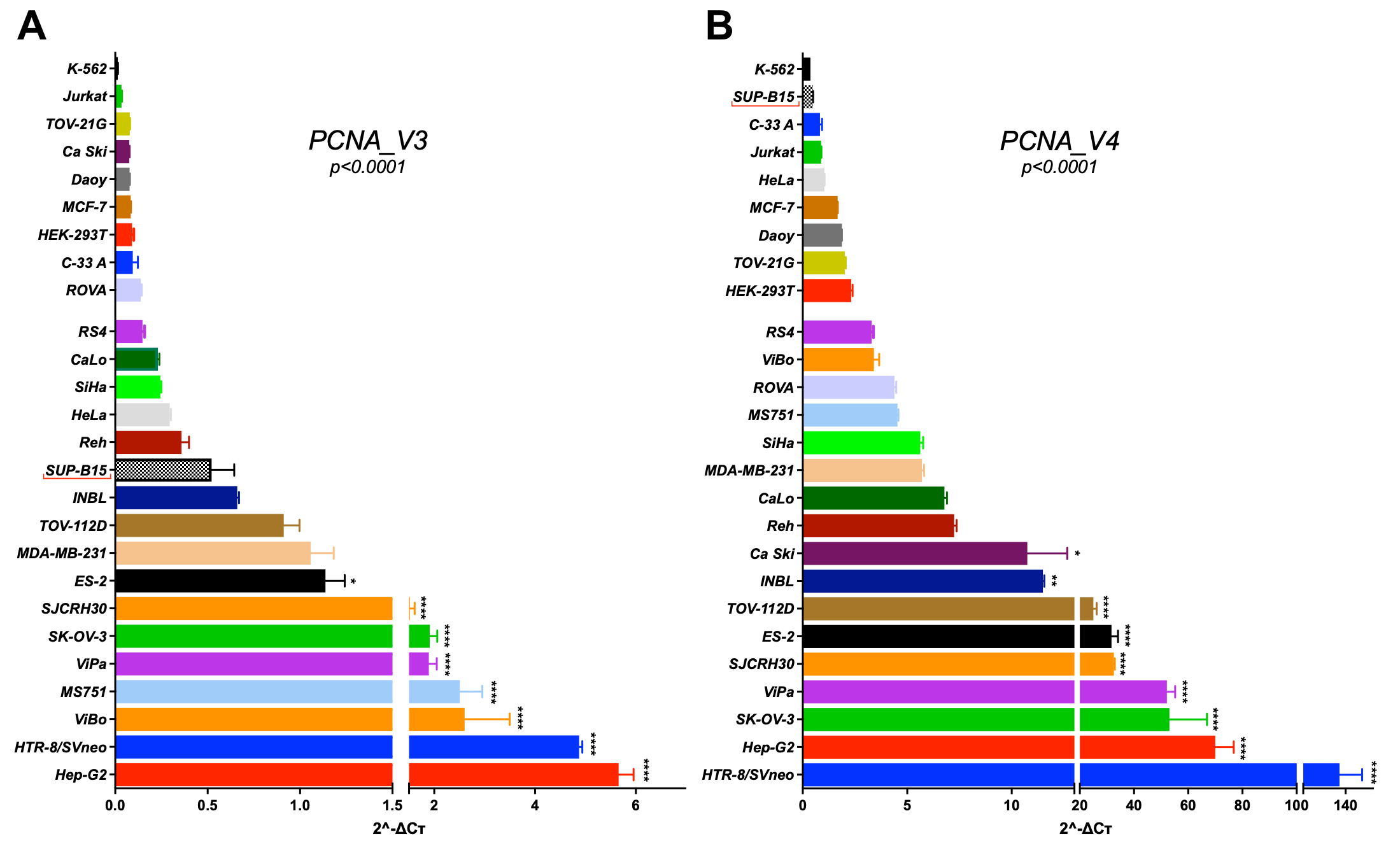

Supplement: Supplementary file 1 [file cells-11-03205-s001.zip › Figure S3.tiff]

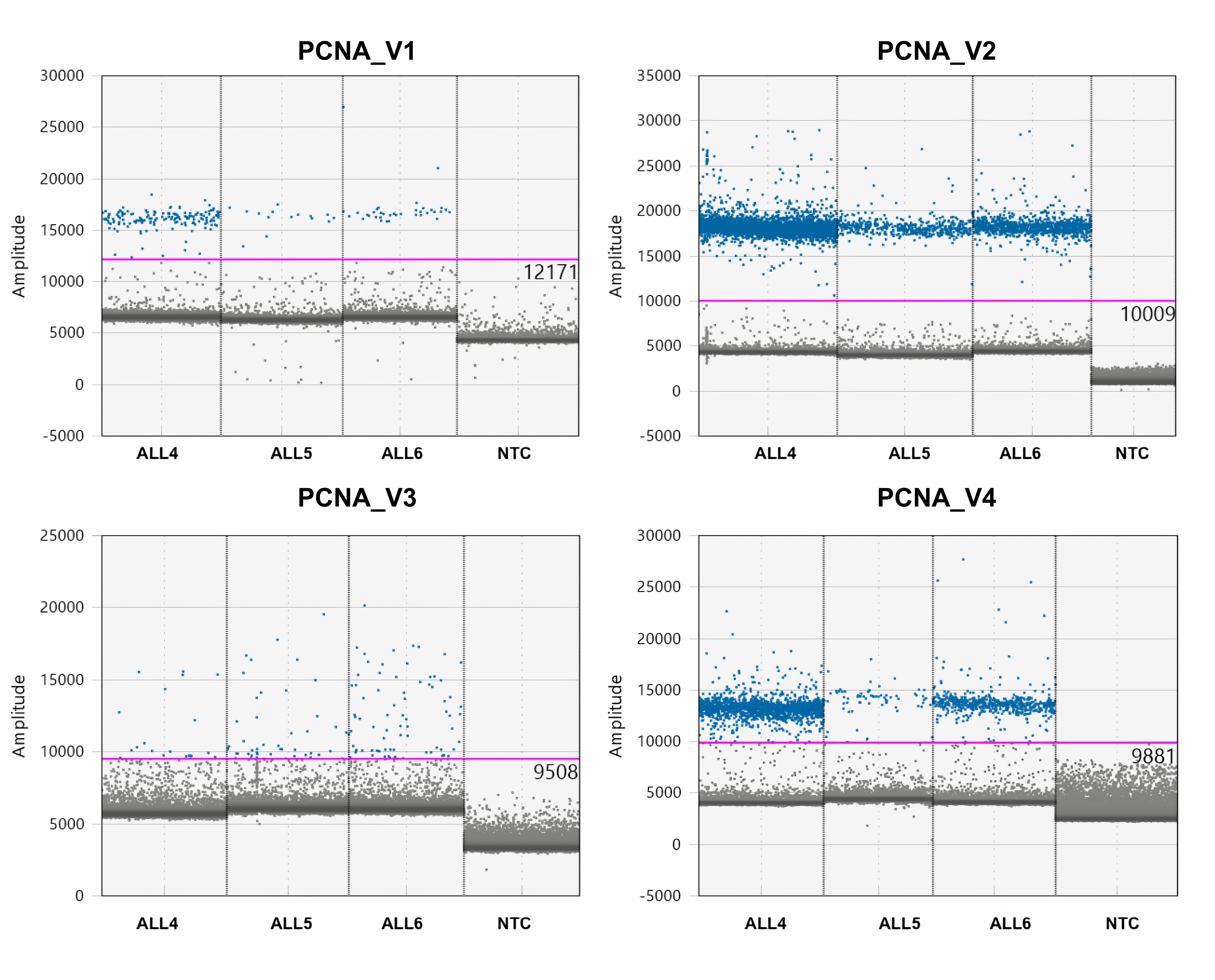

Supplement: Supplementary file 1 [file cells-11-03205-s001.zip › Figure S4.tiff]
